# Hyaluronan synthase 3 mediated oncogenic action through forming inter-regulation loop with tumor necrosis factor alpha in oral cancer

**DOI:** 10.18632/oncotarget.14697

**Published:** 2017-01-17

**Authors:** Yi-Zih Kuo, Wei-Yu Fang, Cheng-Chih Huang, Sen-Tien Tsai, Yi-Ching Wang, Chih-Li Yang, Li-Wha Wu

**Affiliations:** ^1^ Institute of Basic Medical Sciences, College of Medicine, National Cheng Kung University, Tainan 70101, Taiwan, R.O.C; ^2^ Department of Otolaryngology, National Cheng Kung University Hospital, Tainan 70428, Taiwan, R.O.C; ^3^ Department of Radiation Oncology, National Cheng Kung University Hospital, Tainan 70428, Taiwan, R.O.C; ^4^ Department of Pharmacology, College of Medicine, National Cheng Kung University, Tainan 70101, Taiwan, R.O.C; ^5^ Institute of Molecular Medicine, College of Medicine, National Cheng Kung University, Tainan 70101, Taiwan, R.O.C; ^6^ Department of Medical Laboratory Science and Biotechnology, Kaohsiung Medical University, Kaohsiung 80708, Taiwan, R.O.C

**Keywords:** hyaluronan, HAS3, oral cancer, TNF-α, MCP-1

## Abstract

Hyaluronan (HA) is a major extracellular matrix component. However, its role and mediation in oral cancer remains elusive. Hyaluronan synthase 3 (HAS3), involved in pro-inflammatory short chain HA synthesis, was the predominant synthase in oral cancer cells and tissues. HAS3 overexpression significantly increased oral cancer cell migration, invasion and xenograft tumorigenesis accompanied with the increased expression of tumor necrosis factor alpha (TNF-α) and monocyte chemoattractant protein 1 (MCP-1). Conversely, HAS3 depletion abrogated HAS3-mediated stimulation. HAS3 induced oncogenic actions partly through activating EGFR-SRC signaling. HAS3-derived HA release into extracellular milieu enhanced transendothelial monocyte migration and MCP-1 expression, which was attenuated by anti-HAS3 antibodies or a HAS inhibitor, 4-Methylumbelliferone (4-MU). The NF-κB-binding site III at -1692 to -1682 bp upstream from the transcript 1 start site in HAS3 proximal promoter was the most responsive to TNF-α-stimulated transcription. ChIP-qPCR analysis confirmed the highest NF-κB-p65 enrichment on site III. Increased HAS3 mRNA expression was negatively correlated with the overall survival of oral cancer patients. A concomitant increase of TNF-α, a stimulus for HAS3 expression, with HAS3 expression was not only associated with lymph node metastasis but also negated clinical outcome. Together, HAS3 and TNF-α formed an inter-regulation loop to enhance tumorigenesis in oral cancer.

## INTRODUCTION

Hyaluronan (HA), a polysaccharide of repeating units of D-glucuronic acid and N-acetyl-glucosamine in body fluids and tissues, is an essential component of extracellular matrix [1]. HA regulates target cell adhesion, migration and proliferation, and participates in tissue homeostasis and biomechanical integrity [2, 3]. In addition to regulating inflammatory gene expression, immune cell recruitment, and cytokine release [4], HA also promotes tumor angiogenesis [5]. HA is overproduced by many cancer types and, in some cases, the level of HA is prognostic for malignant progression [1, 6]. Increased HA levels may thus provide an environment facilitating various aspects of tumor progression.

HA synthases (HASs) are unique plasma membrane glycosyltransferases. Three related synthases, HAS1, HAS2 and HAS3, synthesize and secrete different sizes of HA polmers directly to the extracellular space in human cells [7, 8]. High-molecular-mass HA (HMM-HA) represses mitogenic signaling with anti-inflammatory properties, whereas low-molecular-mass HA (LMM-HA) promotes proliferation and inflammation [9, 10]. HAS1 with the lowest enzymatic activity maintains a low basal HA level. Although HAS2 is the most studied synthase involved in embryonic and cardiac cushion morphogenesis and can stimulate cell proliferation and angiogenesis, HAS3 is the most active in the synthesis of short chain HA (100~1000 kDa) and highly expressed in tumor cells [7]. Elevated HAS3 expression promoted the growth of prostate, colon and pancreatic cancer cells and angiogenesis in prostate cancer [11–13]. Systemic inhibition of HA synthesis or HAS3 knockdown decreased esophageal xenograft tumorigenesis [14]. In contrast to a potential oncogenic role of HAS enzymes in carcinogenesis, HAS3 under-expression was a poor prognositic marker for bladder cancer [15] and the promoter methylation of HAS3 was recently proposed to regulate hyaluronan production in pancreatic cancer [16].

HA has been associated with tumorigenesis for some time [17]. Overall, about 25-30% of human tumors overexpress HA [18]. HA was also increasingly released in oral cancer patient sera relative to those in patients with benign tumors and in healthy controls [19]. By constrast, the reduction of HA staining was correlated with poor survival in oral cancer patients [20]. HA synthesis is one important mechanism for HA accumulation in tumors [21]. The exact role of HA and the contribution of individual HAS enzymes in oral cancer, therefore, remains to be characterized. The present study was aimed to study the role of the over-represented HAS member by overexpression or knockdown approaches in oral cancer and examine the action mechanism of the HAS-derived HA involved in the crosstalk between oral cancer cells and their microenvironment. Our data support an important role of HAS3-mediated short-chain HA for providing a favorable environment for oral carcinogenesis.

## RESULTS

### Predominant HAS3 expression in most oral cancer cells

**Figure 1:**
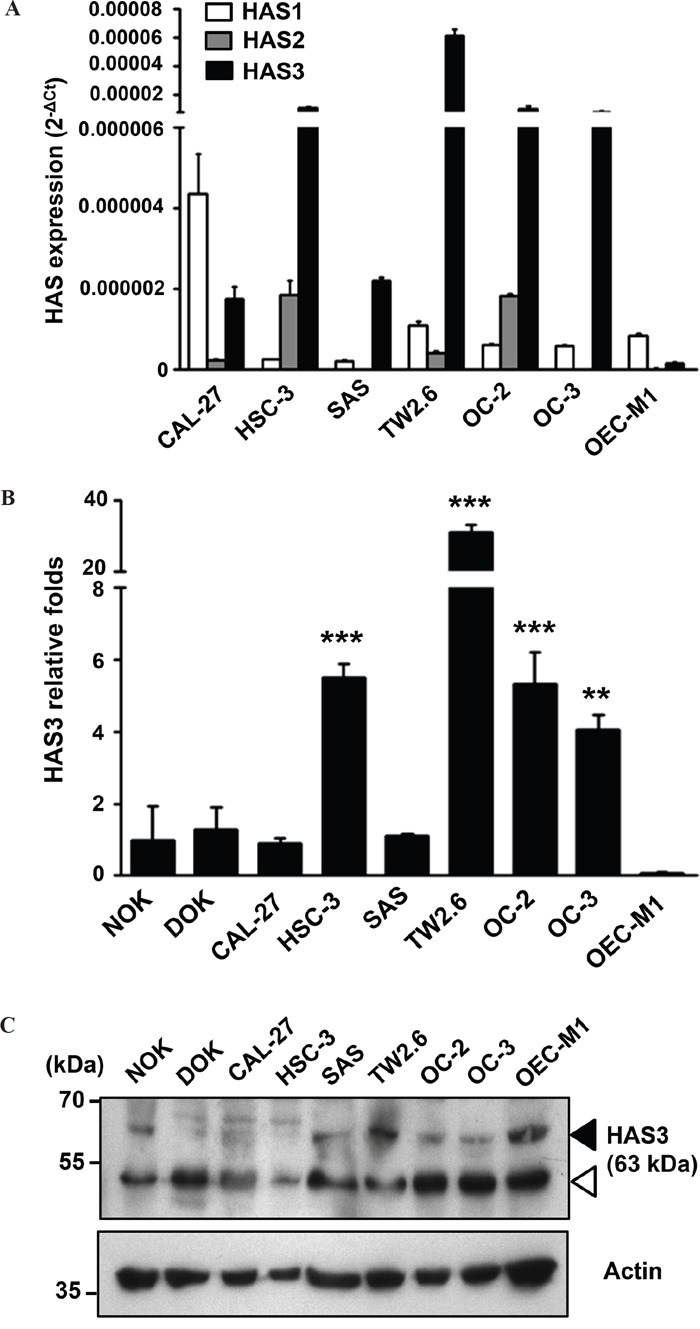
Predominant HAS3 expression in oral cancer cells **A**. The mRNA expression of HAS1, HAS2 and HAS3 in 6 oral cancer cell lines was measured by qRT-PCR and expressed as 2^-ΔCt^. **B**. The relative expression of HAS3 mRNA in each oral cell line was measured by qRT-PCR and expressed as relative fold change (2^-ΔΔCt^) when compared with that in NOK. **p<0.01 or ***p<0.01 versus NOK. **C**. HAS3 protein was measured by Western blot analysis. Actin was a loading control. Black arrowhead, HAS3 protein; white arrowhead, nonspecific band. Results are representative of three independent experiments. ** p<0.01; *** p<0.001 versus NOK; One-way ANOVA and Tukey's multiple comparison test, mean ± SD.

With frequently altered HAS expression in several cancer types [22], we studied if HAS enzymes were also deregulated in oral cancer. Due to 57-71% amino acid identity among HAS members [23] and possible contribution of HAS expression from different cell types [24] in clinical specimens, we measured relative HAS1-3 mRNA expression in 6 oral cancer lines by using qRT-PCR. HAS3 mRNA was most expressed in 5 oral cancer lines (Figure 1A). We further compared the expression in oral cancer cells with normal counterparts, NOK or DOK. Oral cancer cells manifested an increase of HAS3 mRNA and protein expression in most oral cancer lines relative to normal counterparts (Figure 1B-1C). Together, HAS3 was the dominantly expressed HA synthase in most oral cancer cell lines.

### Increased HAS3 expression mainly promoted oral cancer migration and invasion

**Figure 2:**
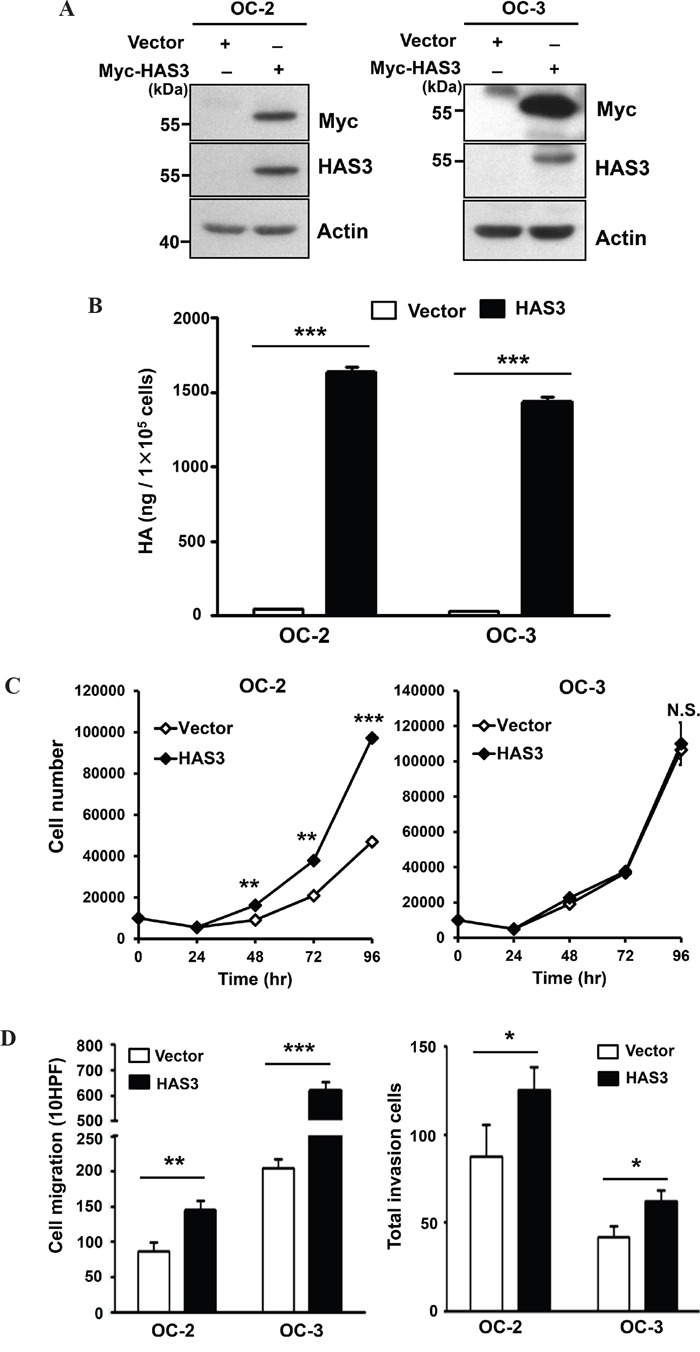
Ectopic HAS3 expression promoted HA accumulation, cell migration, and invasion **A**. Oral cancer cells, OC-2 (Left) or OC-3 (Right) were infected with lentiviruses bearing empty vector or Myc-tagged HAS3. Total cell lysates were harvested for Western Blot analysis of ectopic and endogenous HAS3 proteins. The blot shown is representative of three independent experiments. Actin was a loading control. **B**. HA concentrations in CM derived from the indicated cells were measured by ELISA and expressed as ng per 10^5^ cells. **C**. Viable cell numbers were enumerated by cell proliferation assay. **D**. Cell migration and invasion abilities were measured, respectively, by Transwell migration (Left) and invasion (Right) assays. C and D results are representative of two-three independent experiments performed in triplicate. *p<0.05; ** p<0.01; *** p<0.001; N.S, not significant; Student's t test.

To study the role of HAS3 dergulation in oral cancer, we manipulated HAS3 expression in oral cancer lines, OC-2 and OC-3, followed by using various cell-based transformation assays. We confirmed ectopic Myc-tagged HAS3 as well as total HAS3 protein in these cells by Western blot analysis and the consequent increase of HA accumulation in the CM by ELISA (Figure 2A and 2B). We next examined whether altered HAS3 expression would affect oral cancer cell growth, migration, and invasion. Ectopic HAS3 expression increased OC-2 but not OC-3 proliferation (Figure 2C). In Transwell migration and invasion assays, increased HAS3 expression promoted the migration and invasion of both OC-2 and OC-3 cells (Figure 2D).

**Figure 3:**
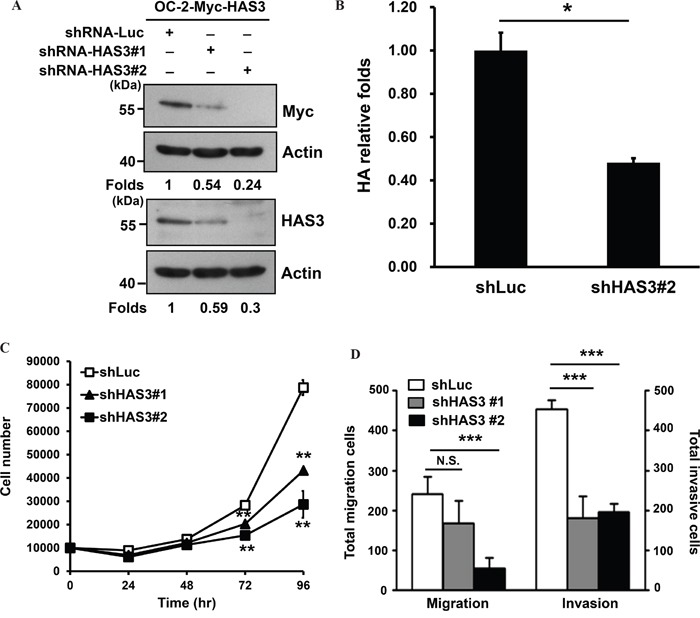
HAS3 silencing reduced HA accumulation, cell proliferation, migration, and invasion **A**. Myc-tagged HAS3-expressing OC-2 cells (OC-2-HAS3) were infected with lentiviruses bearing shLuc or shRNA-HAS3(clone #1 or #2) to deplete HAS3 expression in these cells. Western blot analysis of Myc-tagged HAS3 expression in the indicated knockdown cells. Actin was a loading control. The numbers on the bottom are expression folds relative to shLuc control. This experiment is representative of two biological repeats. **B**. Following HA concentrations in the indicated CM by using ELISA, HA production was expressed as relative folds following normalization with shLuc control cells. *p<0.05; Student's t test. **C**. Viable cells in the control shLuc or HAS3-depeleted OC-2 clones were enumerated by cell proliferation assay. **D**. The migration and invasion abilities of the manipulated cells were measured, respectively, by Transwell migration and matrigel invasion assays. C and D are representative of two independent experiments performed in triplicate. *p<0.05; ** p<0.01; *** p<0.001; N.S, not significant; One-way ANOVA and Tukey's multiple comparison test.

To validate oncogenic promotion exerted by ectopic HAS3 expression, we depleted HAS3 expression in OC-2 expressing Myc-HAS3 cells by transduction with lentiviruses bearing control shLuc or shHAS3 clones (#1 or #2). Western blot and ELISA analyses confirmed the concordant decrease of HAS3 protein expression and HA production in HAS3-depleted cells (Figure 3A-3B). The knockdown reduced not only the proliferation but also the migration and matrigel invasion of OC2 cells (Figure 3C-3D). Endogenous HAS3 silencing also recapitulated the findings of knocking down ectopic HAS3 in oral cancer cells (Supplementary Figure 1). Together, enhanced HAS3 expression primarily promoted oral cancer cell migration and invasion while having cell-type specific effect on cell proliferation.

### Ectopic HAS3 expression promoted xenograft tumorigenesis

**Figure 4:**
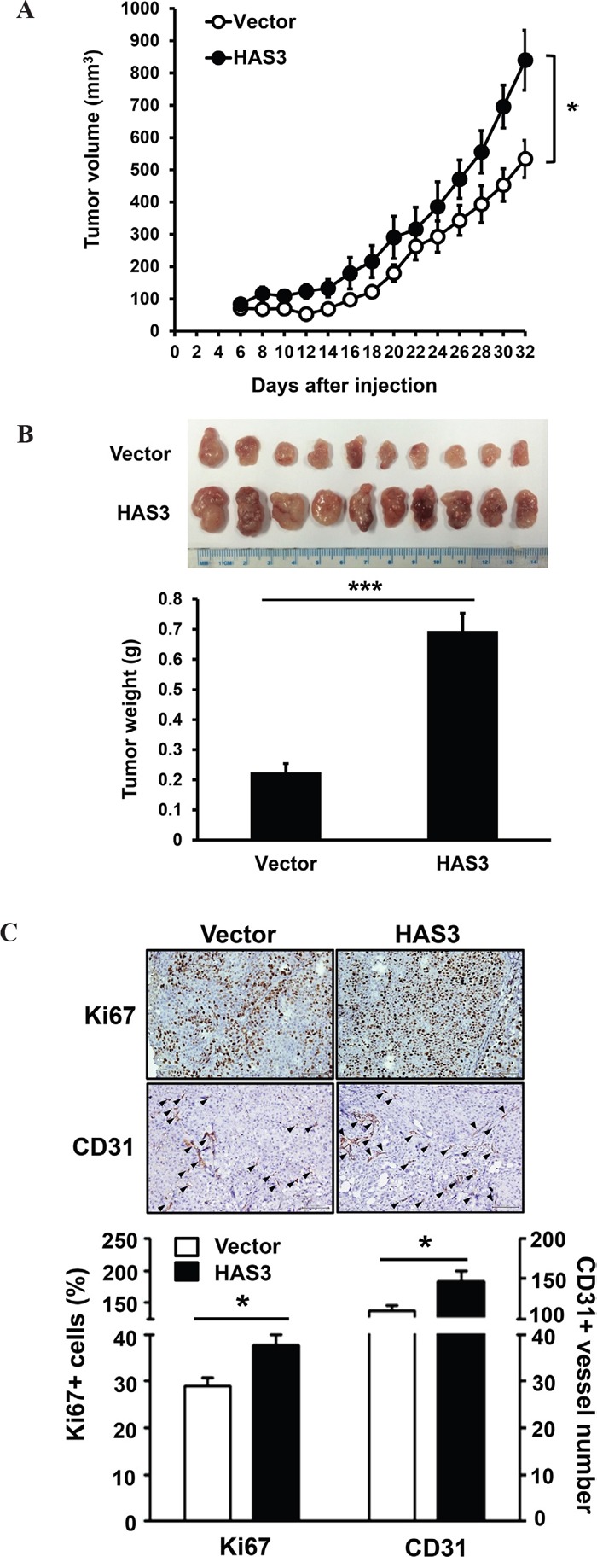
Ectopic HAS3 expression promoted xenograft tumorigenesis *in vivo* **A**. Myc-tagged HAS3- or vector-expressing OC-2 cells were subcutaneously injected into male nude mice (10 mice per group). Tumor volume was measured every 2 days for 32 days. **B**. Tumor images and weights of vector or HAS3 group at the end point. **C**. Top, representative IHC staining of Ki67 and CD31 (arrowhead) in vector or HAS3-OC-2 tumor sections (200X magnification). Bottom, the percentage of Ki67+ nuclei or the number of CD31+ microvessels in five random 200X fields of the indicated mouse tissues were expressed as mean ± SEM. ***p < 0.001; *p < 0.05 versus vector.

To investigate the effect of HAS3 overexpression on xenograft tumorigenesis, we subcutaneously injected vector- or HAS3-expressing OC-2 cells onto male nude mice (10 mice/group) for 32 days. Ectopic HAS3 promoted OC-2 tumor volume and weights compared to vector control (Figure 4A-4B). IHC staining showed a significant increase of proliferating Ki67-positive cells and CD31-positive microvessel numbers in HAS3 tumors relative to vector tumors (Figure 4C). Ectopic HAS3 expression significantly promoted xenograft tumorigenesis.

### Oncogenic HAS3 activated SRC-EGFR signaling axis in oral cancer cells

**Figure 5:**
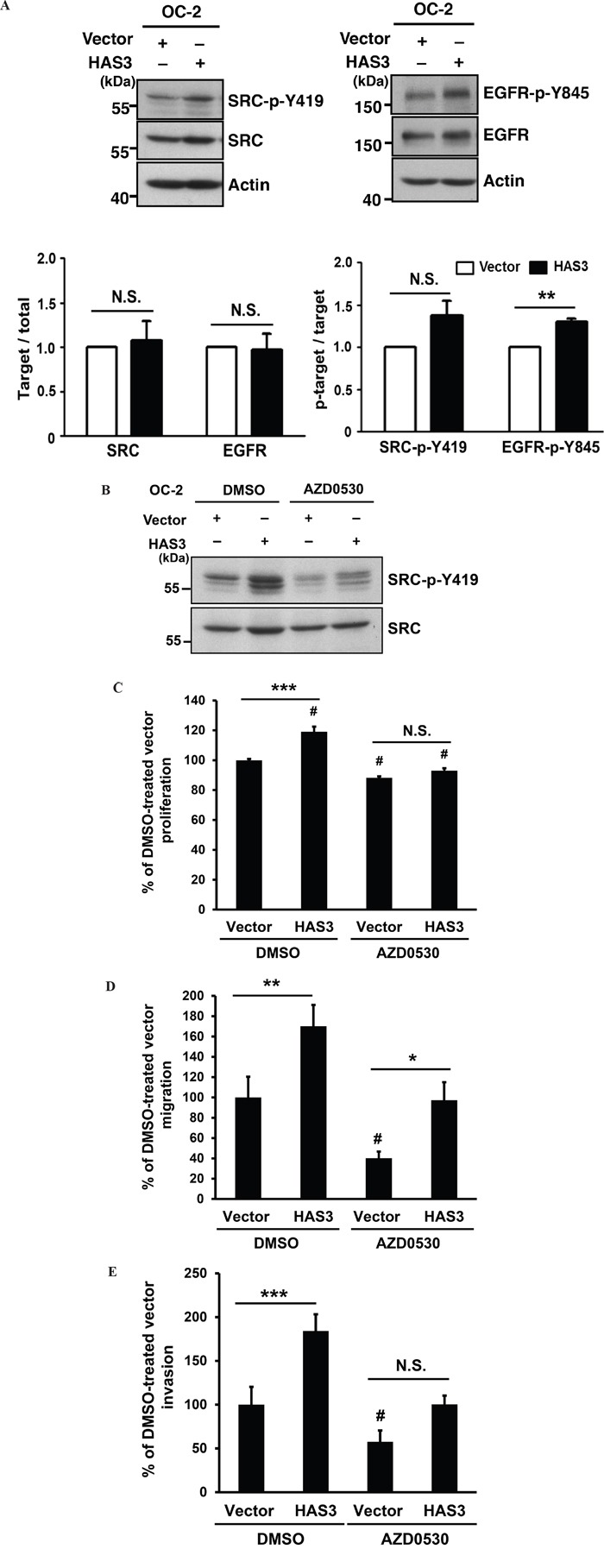
HAS3-mediated oncogenic actions were partly through the activation of Src-EGFR signaling axis **A**. Top, the expression levels of Src and its activating phosphorylation at Y419 residue (Left) or those of EGFR and its activating phosphorylation at Y845 (Right) in the OC-2 cells infected with vector or HAS3-bearing lentiviruses. Actin was a loading control. The blot shown is representative of 2 independent experiments. Bottom, the normalized activating phosphorylation of Src and EGFR in the indicated cells was expressed as mean ± SD (N=3). ** p<0.01; N.S, not significant; Student's t test. **B**. Following DMSO or AZD0530 (2 μM) treatment, the indicated cell lysates were analyzed for Y419 phosphorylation and total level of Src by Western blot analysis. The blot shown is representative of 2 independent experiments. **C-E**. The proliferation, migration and invasion abilities of vector- or HAS3-expressing OC-2 cells in the presence of DMSO (0.1%) or AZD0530 following normalization with DMSO-treated vector control. Results are representative of two independent experiments performed in triplicate. *p<0.05; ** p<0.01; *** p<0.001; N.S, not significant. ^#^p<0.05 versus DMSO-treated vector control. One-way ANOVA and Tukey's multiple comparison test.

Increased HAS3 expression promoted HA production in the CM (Figure 2B). HA binding promotes the activating phosphorylation of c-SRC at tyrosine 419 (Y419) in certain epithelial tumor cells [25]. HA also promotes EGFR-mediated signaling [26] and SRC participates in Y845 phosphorylation of EGFR [27]. To investigate the mechanism for HAS3-mediated oncogenic actions, we used Western blot analysis to examine the impact of HAS3 overexpression on the expression of SRC and EGFR as well as their tyrosine phosphorylation, SRC-Y419 and EGFR-Y845, in OC-2 cells. The increase of HAS3 expression enhanced the levels of SRC-p-Y419 and EGFR-p-Y845 in these cells without affecting the total expression of each protein (Figure 5A). AZD0530 (2 μM), an SRC family inhibitor, attenuated HAS3-mediated increase of SRC-p-Y419 in both vector- and HAS3-expressing cells compared to DMSO control (Figure 5B). Consistent with the basal SRC activation in vector control cells, the attentuation reduced cell proliferation, migration and invasion in not only HAS3-OC-2 but also vector cells (Figure 5C-5E). Together, the activation of SRC-EGFR signaling axis was in part responsible for HAS-3-mediated oncogenic action.

### HAS3-derived CM promoted monocyte recruitment and MCP-1 expression

**Figure 6:**
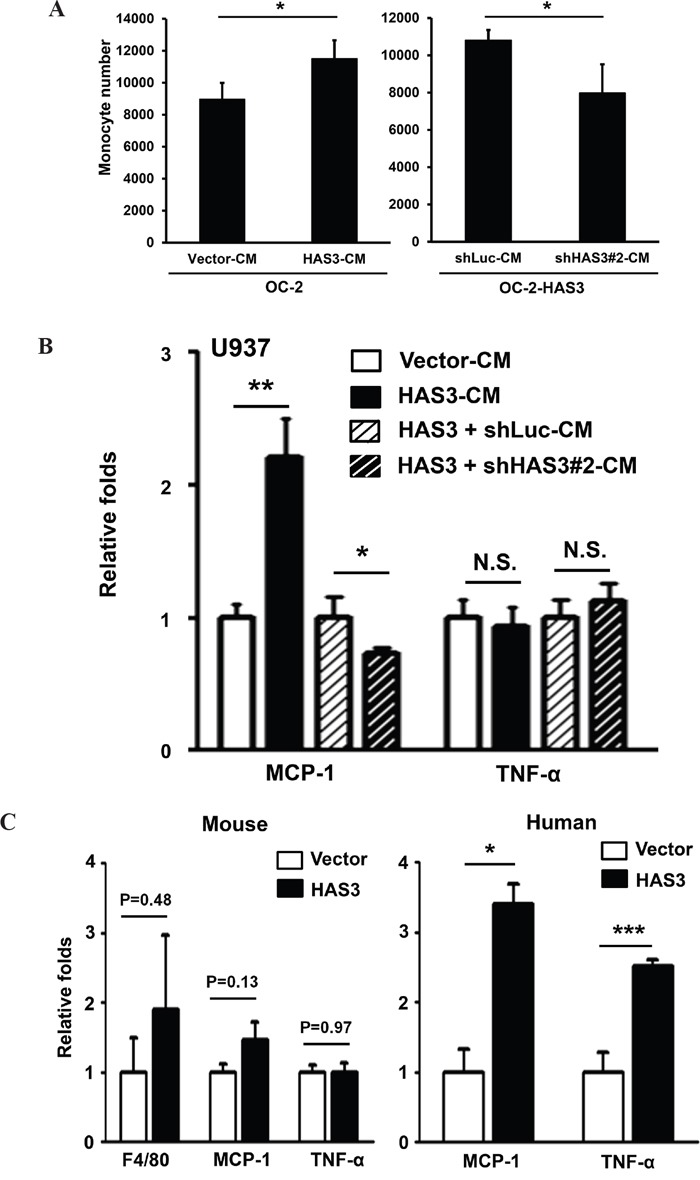
HAS3-altered CM differentially regulated monocyte transendothelial migration and the expression of TNF-α and MCP-1 **A**. Monocytic U937 cell migration through endothelial monolayer was measured after 24-hr incubation with the indicated CM. Left, CM from vector- or HAS3-OC-2 cells; Right, CM from OC-2-HAS3 infected with shLuc or shHAS3 lentiviruses. **B**. The expression of MCP-1 and TNF-α was analyzed in triplicate by qRT-PCR after 48-hr treatment of U937 with the indicated CM and expressed as relative folds. A and B results are representative of two independent experiments performed in triplicate. **C**. The mRNA expression of murine F4/80, MCP-1 and TNF-α in xenograft tumors was analyzed in triplicate by qRT-PCR and expressed as relative folds (Mean ± SEM, N=10). Left, mouse-specific probes. Right, human-specific probes. *p < 0.05; **p<0.01; ***p<0.001;Student's t-test.

HA is implicated in monocyte/macrophage trafficking [4, 5]. With the detected HA increase in HAS3-derived CM relative to vector-CM (Figure 2B), we measured the monocytic U937 transendothelial migration ability mediated by CM. HAS3-CM increased U937 cell recruitment relative to vector-CM, and HAS3 depletion by shRNA#2 reduced the stimulation (Figure 6A). The expression of MCP-1, a regulator for monocyte/macrophage migration and infiltration [28], is inducible by fragmented HA in renal tubular epithelial cells [29]. The recruited monocytes/macrophages can further release pro-inflammatory cytokines like TNF-α to accelerate tumor progression [30]. We analyzed by qRT-PCR if HAS3-CM could affect the expression of MCP-1 and TNF-α in treated U937 cells. The expression of MCP-1 mRNA was significantly up-regulated by HAS3-CM but down-regulated by shHAS3#2-CM when compared with the indicated control. HAS3-altered CM, however, had no effect on TNF-α expression (Figure 6B).

We also examined the *in vivo* effect of enforced HAS3 expression on differential regulation of these two cytokines in xenograft tissues. The contribution of macrophages was studied by analyzing the expression of F4/80, a murine macrophage marker [31]. Despite no stimulation of F4/80, MCP-1 or TNF-α mRNA expression from mouse stroma, the expression of human MCP-1 and TNF-α mRNA was significantly increased in HAS3 xenografts relative to that in vector grafts, suggesting the existence of inter-regulation of HAS3 with cytokines *in vivo* tumors (Figure 6C). Taken together, HAS3-CM increased monocytic transendothelial migration partly through enhanced MCP-1 expression *in vitro* and *in vivo*.

### A requirement of HA accumulation for HAS3-mediated oncogenic action

**Figure 7:**
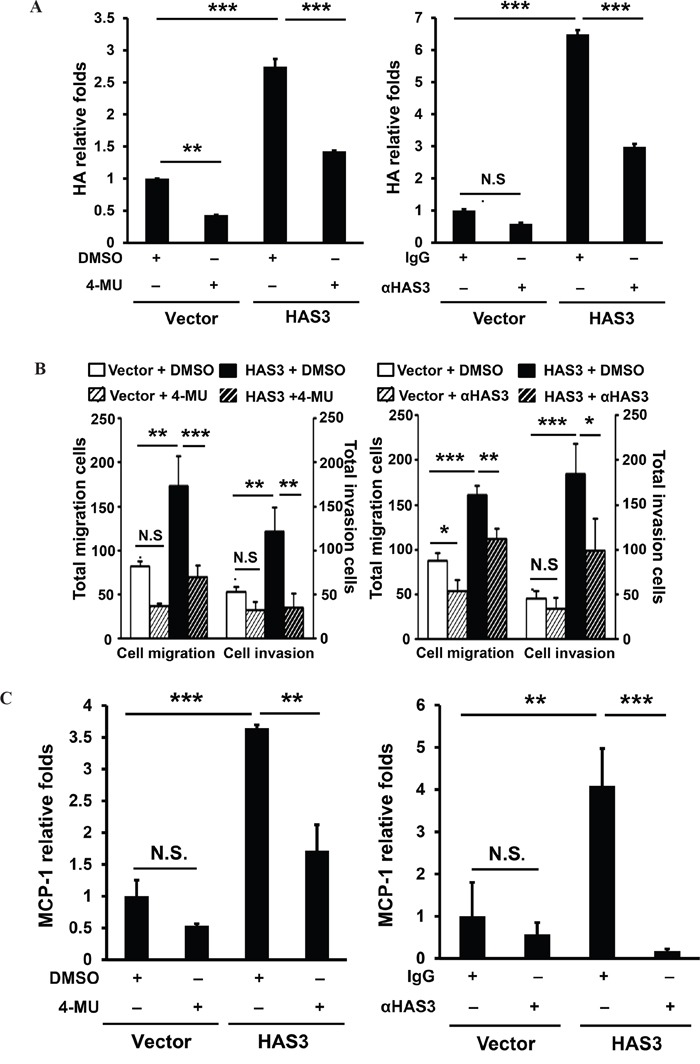
Inhibition of HAS3-mediated HA accumulation reduced the migration, invasion and MCP-1 expression in oral cancer cells Following the inhibition of HAS3 activity by 4-MU (1 mM) or HAS3 antibodies (10 μg/mL) for 6-24 hrs, the treated cells were subjected to **A**. ELISA for HA accumulation, **B**. migration and invasion assays and **C**. for qRT-PCR analysis of MCP-1 expression. HA proudction and MCP-1 expression was expressed as relative folds following normalization with vector control. Results are representative of two independent experiments performed in triplicate. One-way ANOVA and Tukey's multiple comparison test, mean ± SD. *p < 0.05; **p < 0.01; ***p < 0.001.

To examine if HA accumulation in the CM was necessary for the oncogenic action mediated by HAS3, we used pharmacological inhibition or antibody neutralization followed by measuring cell migration, invasion, and monocyte transendothelial migration. MCP-1 mRNA expression in the treated cells was analyzed by qRT-PCR. In addition to reducing HA accumulation (Figure 7A), 4-MU (1 mM) or HAS3 neutralization (10 μg/mL) signficantly attenuated oral cancer cell migration and invasion, and decreased HAS3-mediated increase of relative MCP1 expression in oral cancer cells (Figure 7B-7C). Together, the pharmacological inhibition of HAS enzymatic activity or HAS3 neutralization mimicked HAS3 depletion effects on oral cancer cells, supporting that HA accumulation was accountable for oncogenic actions mediated by HAS3.

### TNF-α-mediated HAS3 transcriptional stimulation through a direct binding of activated NF-κB in oral cancer cells

**Figure 8:**
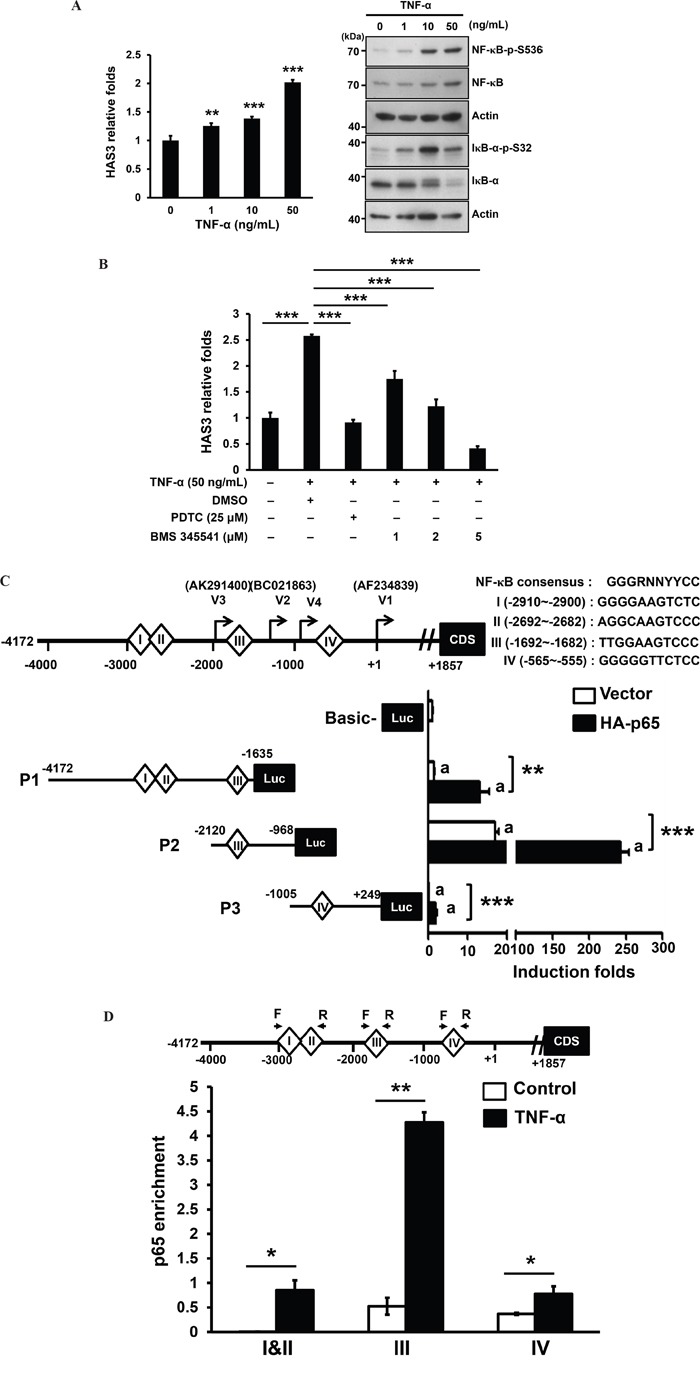
TNF-α mediated transcriptional stimulation of HAS3 expression via direct binding of activated NF-κB **A**. Left, the expression of HAS3 mRNA in OC-2 cells stimulated for 3 hrs with the indicated concentration of TNF-α was measured by qRT-PCR and expressed as relative folds. This experiment is representative of two independent ones. ** p<0.01; *** p<0.001 versus untreated control by ANOVA, Tukey's test. Right, Western blot analysis of NF-κB activating phosphorylation at Ser 536 (NF-κB-p-S536) and IκB-α destabling phosphorylation at Ser32 (IκB-α-p-S32) in OC-2 cells treated for 15 min with TNF-α at 0-50 ng/mL. **B**. Following normalization with untreated control cells, the relative folds of HAS3 mRNA in the oral cancer cells treated for 3 hr with TNF-α (50 ng/mL) in the presence of a NF-κB inhibitor, PDTC (25 μM) or BMS-345541 (1-5 μM). **C**. Top, schematic drawing of the consensus sequence and 4 putative binding sites I-IV for NF-κB binding, and the initiation sites for 4 transcript variants, V1-V4, in the proximal HAS3 promoter spanning from -4172 to +249. This 4-kb promoter was divided into 3 segments, P1-P3, for the luciferase reporter assay. We measured relative luciferase activities in the OC-2 cells tranisently transfected with the indicated promoter constructs, P1-P3, in the absence or presence of NF-κB-p65 subunit (HA-p65). The indicated promoter activity was shown in the bottom as the induction fold following normalization with pGL3-Basic vector activity in oral cancer cells (Mean ± SD of 3 repeats). **p<0.01; ***p<0.001 versus the indicated control. **D**. ChIP-qPCR analysis showed the enrichement of *in vivo* NF-κB-p65 binding to the indicated NF-κB site in P1-P3 promoter region upon TNF-α stimulation for 3 hrs (N=2). *p<0.05; **p<0.01; Student's t test.

TNF-α induced HAS3 mRNA expression in skin fibroblasts and ligament cells with no clear mechanism [32, 33]. We examined by qRT-PCR if TNF-α also stimulated HAS3 mRNA expression in oral cancer cells. OC-2 cells were treated for 3 hrs with recombinant human TNF-α at 1-50 ng/mL prior to total RNA isolation. TNF-α indeed dose-dependently stimulated HAS3 mRNA expression (Figure 8A, Left). Consistent with the inducing role of TNF-α on canonical NF-κB activation [34], TNF-α induced activating phosphorylation of NF-κB-p65 at S536 and destabling phosphorylation of IκBα at S32, leading to NF-κB activation, with a maximal induction at 10-50 ng/mL (Figure 8A, Right). To validate the involvement of NF-κB activation in HAS3 transcription, OC2 cells were co-treated for 3 hr with TNF-α (50 ng/mL) and a NF-κB inhibitor, pyrrolidine dithiocarbamate (PDTC) or BMS-345541. The pharmacological inhibition of NF-κB activation by PDTC (25 μM) or BMS-345541 (1-5 μM) significantly reduced TNF-α-induced HAS3 mRNA expression (Figure 8B), indicating a requirement of canonical NF-κB activation for the transcriptional regulation of HAS3 expression.

Due to the presence of 3 published transcripts (variants 1-3) and one alternative transcriptional initiation (variant 4) [35], the HAS3 promoter spanning -4172 and +249 using variant 1 as transcription start site (+1) was divided into 3 segments, P1-P3, which were individually cloned into pGL3-basic luciferase construct. Four putative NF-κB binding sites, I-IV, were predicted by using LASAGNA-Search 2.0 in this region (Figure 8C, Top). Promoter-driven luciferase assays showed that P2 harbored the highest basal promoter activity and the introduction of NF-κB-p65 subunit (HA-p65) further enhanced the activity of all promoter segments (Figure 8C). To examine if TNF-α could increase the *in vivo* NF-κB binding to the HAS3 promoter in oral cancer cells, we performed ChIP-qPCR analysis of NF-κB sites I and II in P1, III in P2 and IV in P3 promoter regions. Following TNF-α stimulation for 3 hrs, there was an significant increase in the enrichement of *in vivo* NF-κB-p65 binding to each promoter region. The highest enrichement was in site III for NF-κB binding (-1692~ -1682) in the P2 promoter (Figure 8D). Together, TNF-α induced the transcriptional regulation of HAS3 expression through a direct binding of activated NF-κB in oral cancer.

### The increase of both HAS3 and TNF-α expression was correlated with reduced overall surival

**Figure 9:**
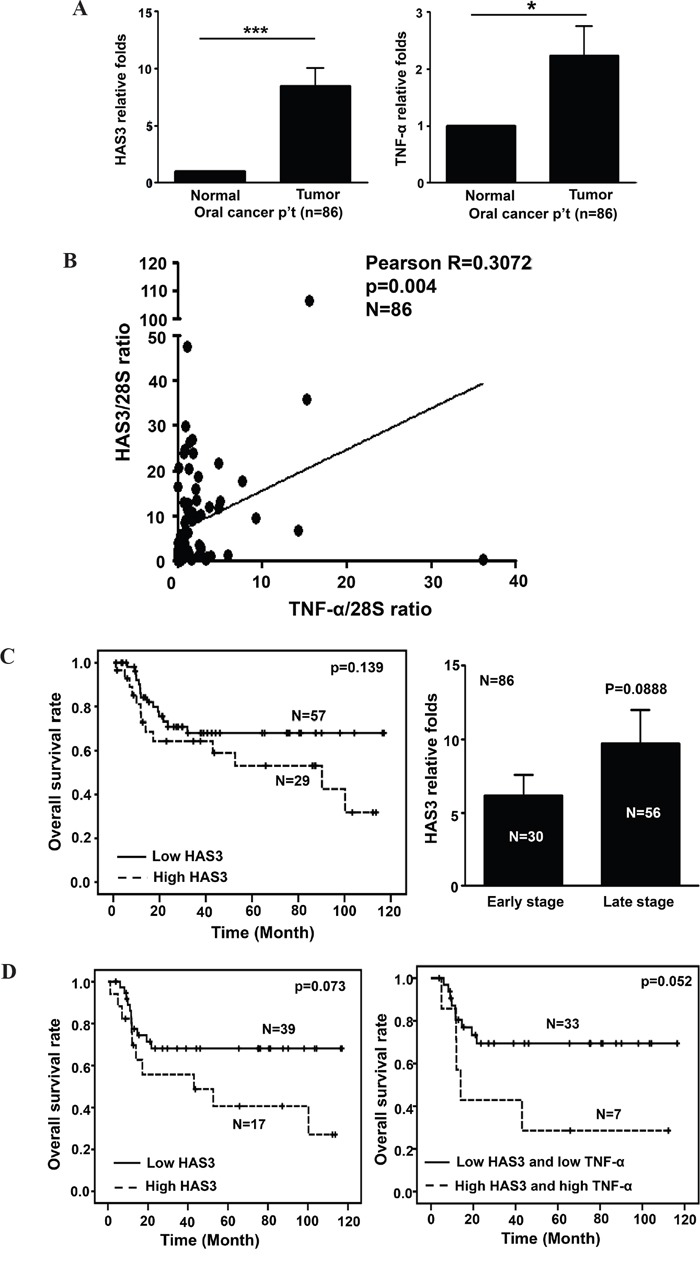
A positive correlation of HAS3 with TNF-α expression in oral cancer **A**. The relative expression of HAS3 (Left) and TNF-α (Right) mRNA in 86 pairwise surgical specimens including tumor and normal tissues was measured by qRT-PCR. Each experiment was carried out in triplicate. Data are Mean ± SEM. **p < 0.01; *p < 0.05 vs normal. **B**. Pearson correlation analysis showing a positive correlation of HAS3 and TNF-α expression in tumor specimens. **C**. Left, Kaplan-Meier survival curve analysis of 86 oral cancer patients with high (> mean) or low (≤ mean) HAS3 expression. Right, the relative folds of HAS3 mRNA expression in early and late stage oral cancer patients. **D**. Left, Kaplan-Meier analysis showed that late-stage oral cancer patients with high expression of both HAS3 and TNF-α expression had reduced overall survival relative to those with both low expression (p = 0.073). Right, the concordant increase of HAS3 with TNF-α expression in oral cancer patients had poorest overall survival (p = 0.052).

In addition to TNF-α-mediated HAS3 transcriptional induction, chronic TNF-α exposure induced oral cancer cell stemness [36]. However, the relationship between HAS3 and TNF-α expression has never been examined in human cancer patients. Due to the inability of having specific HAS3 protein staining in clinical specimens, we, therefore, investigated the transcript levels of HAS3 and TNF-α in pairwise oral cancer specimens by using qRT-PCR. We first validated that HAS3 was also the most expressed HAS member in 30 clinical tissues (Supplementary Figure 2). The expression of HAS3 or TNF-α mRNA was significantly increased in 86 oral cancer relative to adjacent normal tissues (Figure 9A). Pearson correlation showed a positive association in the increase of both expression in the same specimens (Figure 9B, p=0.004). We divided these patients into 2 groups, high (> mean) and low (≤ mean), for its relation with clinicopathologic characteris and Kaplan-Meier survival curve analysis based on the mean HAS3 mRNA expression. Although there was no significant association of HAS3 deregulation with any clinicopathologic characteristics (Supplementary Table 1), high HAS3 patients tended to have poor overall survival relative to low HAS3 ones (Figure 9C, Left, p = 0.139). With the increasingly present of HAS3 mRNA in the late stages (Figure 9C, Right), we examined if increased HAS3 mRNA expression had any impact on the overall survival of these patients. High HAS3 expression further reduced the overall survival for late-stage oral cancer patients (Figure 9D, Left, p = 0.073). Since TNF-α may function as a upstream mediator for HAS3 expression in oral cancer, we next studied the correlation of TNF-α deregulation with late-stage patients’ clinicopathologic characteristics and clinical outcome. We found that high TNF-α expression in high HAS3 patients was mainly associated with the involvement of lymph nodes (Table 1, p =0.008) and further reduced their clinical outcome with a borderline p value of 0.052 (Figure 9D, Right). Together, a concordant increase of HAS3 with TNF-α expression could potentially serve as a poor prognosis signature for oral cancer.

**Table 1:** Mean HAS3 and TNF-α expression in relation to clinicopathologic characteristics of oral cancer

		HAS3/TNF-α expression		P value
		Low/Low	High/High	
	Total (n=59)	47 (79.7 %)	12 (20.3 %)	
**Stage**				
Early (I+II)	21	19 (90.5 %)	2 (9.5 %)	0.125
Late (III+IV)	38	28 (73.7 %)	10 (26.3 %)	
**Tumor status (T)**				
T1-2	37	30 (81.1 %)	7 (18.9 %)	0.725
T3-4	22	17 (77.3 %)	5 (22.7 %)	
**Lymph nodes (N)**				
No	30	28 (93.3 %)	2 (6.7 %)	0.008**
Yes	29	19 (65.5 %)	10 (34.5 %)	
**Distant metastasis (M)**				
No	58	47 (81 %)	11 (19 %)	0.046^a^
Yes	1	0 (0 %)	1 (100 %)	
**Differentiation**				
Well	31	25 (80.6 %)	6 (19.4 %)	0.843
Not well	28	22 (78.6 %)	6 (21.4 %)	
**Recurrence**				
No	41	34 (82.9 %)	7 (17.1 %)	0.347
Yes	18	13 (72.2 %)	5 (27.8 %)	

** p < 0.01 by Chi-square test.

a, only 1 case with distant metastasis.

## DISCUSSION

HA is important for tumor progression in several cancer types [22] and its synthesis is correlated with the expression of HAS family members [37]. In this study, HAS3, the most abundant member in oral cancer cells, was increasingly present in oral cancer tissues compared to their normal counterparts. The increase of HAS3 expression predominantly promoted oral cancer migration and invasion *in vitro* and xenograft tumorigenesis *in vivo*. HAS3 depletion negated the oncogenic actions. SRC-EGFR signaling axis was activated in HAS3-mediated oncogenic actions. HAS3-derived HA accumulation increased transendothelial migration of monocytes possibly through induction of MCP-1 expression. TNF-α, frequently overexpressed in advanced oral cancer tissues [38], functioned as an upstream inducer for HAS3 transcriptional expression partly through NF-κB activation. The increase of both HAS3 and TNF-α mRNA expression was correlated with decreased overall survival for oral cancer patients.

In addition to HAS3, HAS1 or HAS2 was also implicated in human malignancy [39, 40]. Despite the detection of both HAS1 and HAS2 in oral cancer cells, their expression levels were lower than that of HAS3 in most oral cancer cell lines (Figure 1A) not to mention that HAS1 and HAS2 are catalytically less active than HAS3 in HA synthesis [41]. The predominant HAS3 expression was also validated by qRT-PCR analysis in clinical oral cancer specimens regardless of the presence of stromal cells (Supplementary Figure 2). Over-expression and knockdown experiments together with a pharmacological inhibition and anti-HAS3 antibody blockage supported an oncogenic role of HAS3 in oral cancer. Although HAS3 appears to play a dominant role in oral cancer, we still can not rule out the involvement of HAS1 or HAS2 in HA synthesis nor explain why reduced HA staining was a poor prognositic maker in other oral cancer cohort [20]. More studies are needed to examine the differential contribution of HAS1-3 to HA synthesis in oral cancer.

Among many cell surface receptors for HA-mediated signaling [42], HA bound CD44 receptor critically mediates tumor cell growth, survival, migration, and metastasis [43]. Consistent with the HA-mediated activation of SRC-EGFR signaling axis [25, 26], HAS3 overexpression also increased the levels of SRC-p-Y419 and EGFR-p-Y845. Pharmacological reduction of SRC activity by AZD0530 significantly impaired HAS3-mediated promotion of oncogenic action. We also detected a negative effect of AZD0530 on vector control cells. The reason could be twofold. First, vector OC2 cells had constitutive SRC activation (Figure 5A) as well as endogenous HAS3 expression (Figure 1C). Second, AZD530 inhibited not only SRC but also its related kinases like ABL [44]. More studies are needed to verify if CD44 is the sole receptor for HA and the involvement of additional SRC-related kinases in HAS3-mediated oncogenic signal axis in oral cancer.

The amount of HA was altered accordingly in the CM derived from HAS3-manipulated oral cancer cells (Figure 2B and 3B). Fragmented HA induced MCP-1 expression in renal epithelial cells [29]. A concordant expression of MCP1 with HAS3 in HAS3-manipulated oral cancer cells further supported a positive role of HAS3 on MCP1 expression. LMM-HA could activate macrophages via enhanced TNF-α secretion [45] or increased TNF-α expression in chondrocytes [46]. In addition to the production of pro-inflammatory short chain HA by HAS3 [1, 7], the expression of HAS3 was regulated by several cytokines including TNF-α in nonepithelial cells [33]. In contrast to MCP1, we only detected human TNF-α increase in the HAS3 xenograft tissues but not in HAS3-derived CM-treated monocytes (Figure 6B and 6C). The increased TNF-α expression was also observed in HAS3-overexpressing oral cancer cells by qRT-PCR (data not shown). The inter-regulation of HAS3 and cytokines adds another layer of complexity in reglating HAS3 expression in cancer.

Constitutive TNF-α production is a characteristic for many malignant tumors [47], and linked to all steps of tumorigenesis in many cancer types [48]. Consistent with a positive role of TNF-α on NF-κB activity [49] and a potential mediation of HAS3 expression by TNF-α, we detected a dose-dependent increase of HAS3 mRNA expression and canonical NF-κB activation in TNF-α-treated oral cancer cells. The pharmacological inhibition of NF-κB activity significantly impaired the stimulation by TNF-α. The *in vivo* binding of NF-κB-p65 was highly enriched on the site III (-1692~-1682) in the most active P2 promoter that is identical to the recently identified HAS3 core promoter [35]. This study confirmed for the first time that TNF-α with its downstream effector NF-κB activity is a potential inducer for HAS3 overexpression in oral cancer.

TNF-α is normally not detectable in plasma or serum of healthy individuals but can be detected in some cancer patients, almost invariably those with advanced disease and poor prognosis [47]. Consistent with TNF-α being an upstream mediator for HAS3 expression, the expression of HAS3 was positively correlated with that of TNF-α in oral cancer specimens. Despite no significant correlation of increased HAS3 expression with reduced patient clinical outcome or with any clinicopathologic characteristic, a concordant increase of both TNF-α and HAS3 expression not only was associated with lymph node metastasis but also further reduced overall survival among late-stage oral cancer patients. Since other cytokines also regulate HAS3 expression [33], their contribution to increasing HAS3 expression in oral cancer remains to be studied.

**Figure 10:**
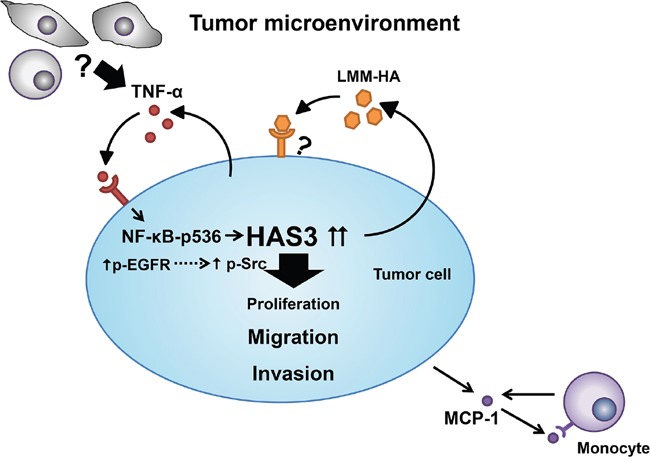
A schematic model for HAS3-mediated oncogenic signaling in tumor microenviroment The binding of HA secreted by HAS3 to cell surface receptors predominantly enhances cell migration and invasion by triggering EGFR-Src signaling axis in tumor cells. Tumor cell-derived HA stimulates MCP-1 release, and recruites monocytes to the microenvironment. Within the tumor microenvironment, pro-inflammatory TNF-α from tumor cells or other stromal cells drives the expression of HAS3 in tumor via NF-κB activation.

In summary, HAS3-mediated HA behaved as a stimulus to trigger intracellular signal transduction pathways, which converge to promote biological activities necessary for cell migration and invasion in oral carcinogenesis. TNF-α and HAS3 formed an inter-regulation loop in mediating oral cancer malignancy via NF-κB activation (Figure 10). Understanding the inter-regulation of HA, TNF-α, and HAS3 in oral cancer might facilitate the development of novel therapeutics for treating this disease and many other critical diseases associated with HA deregulation.

## MATERIALS AND METHODS

### Antibodies and reagents

Keratinocyte serum-free medium (KSFM), other culture media, fetal bovine serum (FBS), Lipofectamine 2000, TRIzol and qRT-PCR reagents were from ThermoFisher Scientific (Grand Island, NY, USA). Oligonucleotide primers for sequencing and qRT-PCR (Table 2) were from MDbio (Taipei, Taiwan). CellTiter 96® AQueous One Solution (MTS kit) and Dual Luciferase reporter assay were from Promega (Madison, WI, USA). pLKO_AS2.zeo was from National RNAi Core facility in Academia Sinica, Taiwan. The 24-well Millicell culture inserts were from Millipore EMD (Darmstadt, Germany). Anti-CD31 antibodies were from Abcam (Cambridge, MA, USA). Matrigel was from BD Biosciences (Bedford, MA, USA). Recombinant human TNF-α was from Peprotech (Rehovot, Israel). PDTC and BMS-345541 were from Tocris Bioscience (Avonmouth, Bristol, UK). The sources and clone names for the used antibodies were listed in Supplementary Table 2.

**Table 2:** List of primers and their sequence for qPCR analysis

Gene	Primer sequence	
	Human	Murine
**HAS1**	F :5’-GGAGATTCGGTGGACTACG-3’	
	R: 5’-TAGGAAGCTGACCCAGGAG-3’	
**HAS2**	F: 5’-CATGTACACAGCCTTCAGAG-3’	
	R: 5’-GGCTGGGTCAAGCATAGTG-3’	
**HAS3**	F: 5’-ATCCAGGTGTGCGACTCTG-3’	
	R: 5’-CACACAGCCAAAGTAGGAC-3’	
**TNF-α**	F: 5’-CCCAGGGACCTCTCTCTAATC-3’	F: 5’-GCCTCTTCTCATTCCTGCTTG-3’
	R:5’-ATGGGCTACAGGCTTGTCACT-3’	R: 5’-GATGATCTGAGTGTGAGGGTCT-3’
**MCP-1**	F: 5’-GCCTCCAGCATGAAAGTCTC-3’	F: 5’-AACTCTCACTGAAGCCAGCTCT-3’
	R: 5’-AGGTGACTGGGGCATTGAT-3’	R : 5’-CGTTAACTGCATCTGGCTGA-3’
**F4/80**		F: 5’-AGCATCCGAGACACACACAG-3’
		R: 5’-GGCAAGACATACCAGGGAGA-3’
**NF-κB II (-2692 ~ -2682)**	F: 5’-GACTGAGGTGTGAGGTCTG-3’	
	R: 5’-TCTCGCAAGTTTTAGGGGCT-3’	
**NF-κB III (-1692~ -1682)**	F: 5’-TTCCTTGCAGGGTGAGTAG-3’	
	R: 5’-ACCTAGAACCTGAAGCGTC-3’	
**NF-κB IV (-565~ -555)**	F: 5’-TTTCGGAGGCTTGATGGAG-3’	
	R : 5’-CCAGCCCAGGCAAACTAAC-3’	

### Patient specimens

Oral cancer specimens were from 86 treatment-naive patients (median age = 52) with 7 females and 79 males undergoing surgery at NCKU hospital. Written informed consent was provided by all participants. With the patients’ informed consent, the biopsies of histologically proven normal tissue in oral cavities other than tumor sites were taken as pair-wised normal controls. All the fresh samples were handeled anonymously, snap-frozen and stored in liquid nitrogen until use. This study was conducted with the approval of the Institutional Review Board at National Cheng Kung Uniersity. All participants gave consent to participate the study and for publication.

### Cell culture

Normal oral keratinocytes (NOK) from gingival tissues of healthy individuals with informed consent were grown in KSFM [50]. Displastic oral keratinocytes (DOK), one gingival cancer line, OEC-M1, two tongue cancer lines, CAL-27 and HSC-3, and three buccal mucosa cancer lines, OC-2, OC-3, and TW-2.6 were maintained as described [51, 52]. Tongue cancer SAS line (RCB1974) was acquired from RIKEN BioResource Center. Human 293T and monocytic U937 cells were maintained as described by American Tissue Culture Collection.

### Plasmid constructs

The HAS3 coding sequence-bearing PCR product spanning from nucleotides 225 to 1886 (Genebank Accession number NM_001199280.1) was cloned into pcDNA3.1(-)/Myc-His A (Invitrogen) and sequence-verified. The Myc/His-tagged HAS3 cDNA fragment was then cloned into pLKO-AS2.zeo lentiviral vector.

### HAS3-expressing stable clone establishment

Vector and HAS3-bearing lentiviruses were prepared from transfection of human 293T cells, respectively, with pLKO-AS2.zeo (Vector) or pLKO-AS2.zeo-HAS3 plasmid using Lipofectamine 2000. Viral particles were collected at 48 hrs post-transfection. Following infection with the indicated lentiviruses at the multiplicity of infection (MOI = 2), stable vector or HAS3-expressing cells were enriched with zeocin (200 μg/mL) for 1-2 weeks before the indicated experiments.

### HAS3 knockdown in oral cancer cells

HAS3-expressing OC-2 cells were established by infection with shRNA-bearing lentiviruses targeting to Luc (control) or HAS3 sequence (MOI = 2). The shRNA clones targeting to HAS3 protein coding sequence, GCTCTACAACTCTCTGTGGTT (TRCN0000045408, clone#1) or CCATTGCTACCATCAACAAAT (TRCN0000045409, clone#2), were from a human library TRC-Hs 1.0 from RNAi Consortium.

### qRT-PCR

Total RNA was isolated by using TRIzol from the indicated cells or snap-frozen tissues. One μg RNA was reverse-transcribed into cDNA using High Capacity cDNA Reverse Transcription Kit (Applied Biosystems, Foster City, CA, USA). We amplified cDNA samples in triplicate by using SYBR Green PCR Master Mix (Roche, West Sussex, UK) and determined the cycle threshold (Ct). Target mRNA expression was calculated by using 2^-ΔCt^ (ΔCt = Ct_target gene_-Ct_28S rRNA_) following the normalization of 28S rRNA as an internal control. When comparing target mRNA expression in two different samples, we used 2^-ΔΔCt^ as relative fold change as described [53].

### Western blot analysis

Cells were lysed in boiled lysis buffer containing 1% SDS and 10 mM Tris–HCl (pH 7.4). Following concentration measurement by Bradford Protein Assay (Bio-Rad Laboratories, Hercules, CA, USA), equal amounts of total cellular protein were fractionated by SDS-PAGE and blotted onto a PVDF membrane. The blot was probed with the indicated primary antibody followed by a horseradish peroxidase-conjugated secondary antibody in the blocking buffer containing 20 mM Tris-HCl (pH7.5), 150 mM NaCl, 0.1% Tween-20, and 5% non-fat milk. For detecting protein phosphorylation, the milk was replaced with 3% bovine serum albumin. The dilution and clone information of antibodies used for hydrization was in Supplementary Table 1. The immunocomplex was detected by Immobilon Western Chemiluminescent HRP substrate (Darmstadt, Germany).

### CM preparation

Subconfluent cells were refed with serum-free culture medium one day after seeding. CM was harvested at 24 hrs after incubation and centrifuged at 3000 rpm to remove cell debris. We used Vivaspin 6 columns (5 kDa MWCO, GE Healthware, Piscataway, NJ, USA) to concentrate the CM.

### Cell proliferation assay

The indicated cells were seeded in triplicate at 10-20% confluence in 24-well plates. Viable cells were numerated daily by trypan blue exclusion for 4 days post-seeding. Alternatively, the cells were quadruplicately seeded at 6000/well for 24 hrs in 96-well plates followed by CM treatment for 48 hrs. Proliferating cells were measured by MTS kits. We independently repeated this experiment 3 times.

### Trans-well migration or invasion assay

These assays were performed in triplicate using 24-well Millicell culture inserts with 8 μm-pore polycarbonate membranes. The indicated cells (3×10^5^/well) were added to upper chambers with membranes coated or not coated with 100 μg of Matrigel. Lower chambers were filled with 500 μL of growth medium. Uncoated filters were used to measure cell migration ability for 6 hrs, whereas coated filters were used for cell invasion for 24 hrs. After 6–24 hrs incubation, the cells that migrated to the lower surface were stained with Giemsa solution and counted in high power fields under a microscope. Both assays were independently repeated two times.

### Xenograft tumorigenesis and IHC staining

The use of male BALB/cAnN.Cg-Foxnlnu/CrlNarl mice (6-8 weeks old) from National Laboratory Animal Center in Taiwan were approved by the Institutional Animal Care and Use Committee. Vector- or HAS3-expressing OC-2 cells (2×10^6^) with 50 µg Matrigel were subcutaneously injected into the flanks of nude mice (10 mice/group). One week after injection, tumor sizes were measured every 2 days for 32 days. Tumor tissues were harvested at the endpoint for IHC staining and RNA/protein isolation following weight measurement. The numbers of Ki67+ and total nuclei as well as CD31+ microvessels in 5 random fields were counted by using Image J software.

### HA measurement by ELISA

The HA concentrations in CM were measured in triplicate by Hyaluronan DuoSet ELISA kits (R&D systems, Minneapolis, MN, USA) with two independent repeats.

### Transendothelial migration of monocytes

HMEC-1 cells (1.5×10^5^/well) seeded in triplicate on collagen-coated Millicell culture inserts were grown to confluence for 3 days. Monocytic U937 cells (10^4^/well) were seeded onto the inserts with TNF-α-activated or untreated endothelial monolayer (a negative control) and allowed to migrate for 24 hrs to bottom wells. The living cells in bottom wells were counted by trypan blue exclusion assays under a light microscope.

### Cytokine treatment

The indicated cells (5×10^5^/dish) were plated overnight in 35mm dishes in RPMI-1640 supplemented with 10% FBS. The cells were serum-deprived for 24 hrs and then treated for 3 hrs with the indicated cytokine for 3 hrs followed by total RNA isolation.

### HAS3 inhibition or neutralization

The indicated cells were treated for 6-24 hrs with the neutralizing HAS3 antibody (10 μg/mL), control IgG (10 μg/mL), 4-MU (1 mM) or 0.1% DMSO (vehicle control) prior to cell-based assays or RNA isolation for RT-PCR analysis.

### Promoter-driven luciferase reporter assay

Following seeding 10^5^ cells/well in triplicate in 24-well plates for 16-18 hrs, cells were transfected by using Lipofectamine 2000 with the indicated promoter construct (400 ng) and pRL-TK (20 ng) as transfection efficiency control with or without 100 ng HA-p65 (from Karin M at University of California, San Diego). Luciferase activities were measured using Dual-Luciferase Reporter Assays and normalized to Renilla luciferase activity at 48 hrs post-transfection.

### Chromatin immunoprecipitation and qPCR (ChIP-qPCR)

ChIP was performed with EZ-ChIP kits (Millipore, Darmstadt, Germany). Briefly, oral cancer cells were serum-deprived for 24 hours and then treated for 3 hrs with 50 ng/ml of TNF-α for 3 hrs. We performed DNA cross-linking by adding 1% formaldehyde into cell cultures for 10 min, followed by neutralization for 5 min with glycine at 0.125 M. Cells were lysed with a lysis buffer with protease inhibitors and sonicated to shear genomic DNA to the lengths in 100-800 bp. The chromatin fragments were immunoprecipitated with isotypic control IgG or anti-NF-κB antibodies. We detected ChIP DNA by qPCR and the primers for NFκB sites (Table 1). Following normalizing ChIP samples with their corresponding input chromatin (ΔCt), the enrichment was defined as change in Ct in treated versus untreated control samples (ΔΔCt), relative to IgG control.

### Statistical analysis

Statistical analyses were performed using a oneway analysis of variance (ANOVA) to compare all pairs of experimental groups. Data were represented as mean ± SD or SEM. p <0.05 was regarded as statistically significant. For comparison of two groups, two-tailed student's t tests were used. All the statistical analyses were performed using Graphpad Prism software. We used linear regression and Pearson correlation to assess the relationship between HAS3 and TNF-α expression. We also used Pearson Chi-square to detect a relationship between clinicopathologic characteristics and HAS3 or TNF-α expression. The Kaplan-Meier method and log-rank test were used to compare the survival among high and low patient groups of HAS3 or TNF-α.

## SUPPLEMENTARY FIGURES AND TABLES


