# Supplementary material for: Hyaluronan synthase 3 mediated oncogenic action through forming inter-regulation loop with tumor necrosis factor alpha in oral cancer
Source: Oncotarget. 2017 Jan 17;8(9):15563–83. doi: 10.18632/oncotarget.14697 (PMC5362506; doi:10.18632/oncotarget.14697)
Supplement: Supplementary file 1 [file oncotarget-08-15563-s001.pdf]

Hyaluronan synthase 3 mediated oncogenic action through forming inter-regulation loop with tumor necrosis factor alpha in oral cancer

SUPPLEMENTARY FIGURES

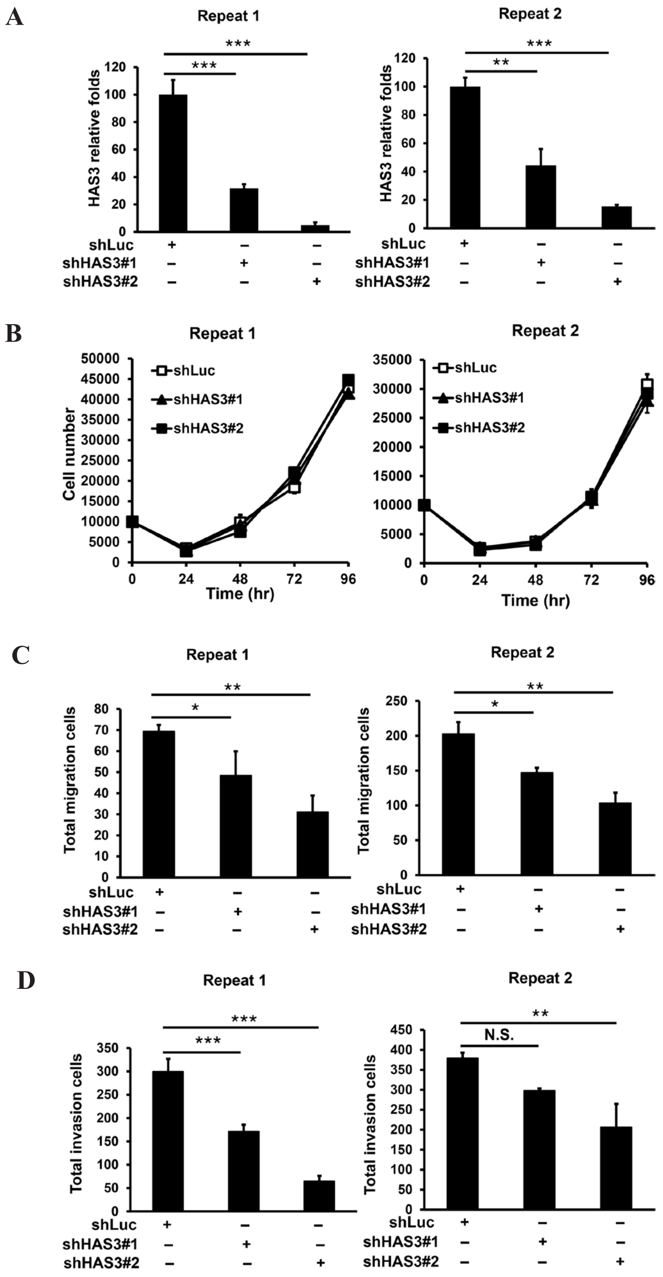

Supplementary Figure 1: Endogenous HAS3 silencing reduced TW2.6 oral cell migration and invasion but not cell proliferation. A. TW2.6 was infected with lentiviruses bearing shLuc or shRNA-HAS3 (clone#1 or #2) to deplete HAS3 expression. The mRNA expression of HAS3 in the indicated knockdown cells by qRT-PCR. **B.** Viable cells numbers were enumerated by cell proliferation assay. **C, D.** The migration and invasion abilities were measured, respectively, by Transwell migration and Matrigel invasion assays. Results are two independent repeats performed in triplicate. *$p<0.05$; ** $p<0.01$; *** $p<0.001$; N.S, not significant versus shLuc, One-way ANOVA and Tukey's multiple comparison test, mean ± SD.

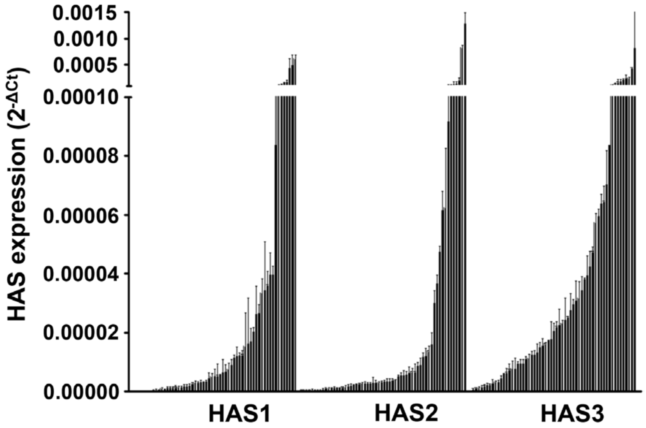

Supplementary Figure 2: Differential expression of HAS1-3 mRNA in oral cancer specimens. Waterfall plots show the relative expression of HAS1, HAS2 and HAS3 mRNA in 30 pairwise surgical specimens including tumor and normal tissues measured by qRT-PCR and expressed as $2^{-\Delta Ct}$.

Supplementary Table 1: Mean HAS3 expression in relation to clinicopathologic characteristics of oral cancer

		HAS3 expression		P value
		Low	High	
	Total (n=86)	57 (66.3 %)	29 (33.7 %)	
**Stage**				
Early (I+II)	30	21 (70 %)	9 (30 %)	0.593
Late (III+IV)	56	36 (64.3 %)	20 (35.7 %)	
**Tumor status (T)**				
T1-2	54	37 (68.5 %)	17 (31.5 %)	0.568
T3-4	32	20 (62.5 %)	12 (37.5 %)	
**Lymph nodes (N)**				
No	42	31 (73.8 %)	11 (26.2 %)	0.149
Yes	44	26 (59.1 %)	18 (40.9 %)	
**Distant metastasis (M)**				
No	85	57 (67.1 %)	28 (32.9 %)	0.158
Yes	1	0 (0 %)	1 (100 %)	
**Differentiation**				
Well	44	27 (61.4 %)	17 (38.6 %)	0.324
Not well	42	30 (71.4 %)	12 (28.6 %)	
**Recurrence**				
No	63	40 (63.5 %)	23 (36.5 %)	0.366
Yes	23	17 (73.9 %)	6 (26.1 %)	

Supplementary Table 2: List of used antibodies and their conditions for experiments

Antibody	Source	Clone	Dilution	Experiment name
Anti-HAS3	Sigma		1:250	Western blot
Anti-Myc	ThermoFisher Scientific	Myc.A7	1:5000	Western blot
Anti-Actin	Millipore	C4	1:10000	Western blot
Anti-Ki67	Leica Biosciences		1:50	Immunohistochemistry
Anti-CD31	Abcam		1:100	Immunohistochemistry
Anti-SRC-p-Y419	R&D systems		1:1000	Western blot
Anti-SRC	Cell Signaling Technology	32G6	1:1000	Western blot
Anti-EGFR-p-Y845	Cell Signaling Technology		1:1000	Western blot
Anti-EGFR	Cell Signaling Technology		1:1000	Western blot
Anti-NF-κB-p-S536 (p65)	Cell Signaling Technology	93H1	1:1000	Western blot
Anti-NF-κB (p65)	Cell Signaling Technology	D14E12	1:1000 / 1:100	Western blot/ChIP
Anti-IκBα-p-S32	Cell Signaling Technology	14D4	1:1000	Western blot
Anti-IκBα	Cell Signaling Technology	L35A5	1:1000	Western blot
Anti-HAS3	Santa Cruz Biotech	E15	10 µg/ml	Co-culture
